# Griffiths Scales of Child Development 3rd Edition: normalization for the Brazilian population

**DOI:** 10.3389/fped.2025.1481442

**Published:** 2025-02-10

**Authors:** Amanda Tragueta Ferreira-Vasques, Eduardo Pimentel da Rocha, Elizabeth Green, Dionísia Aparecida Cusin Lamônica

**Affiliations:** ^1^ Laboratory for the Investigation of Neurodevelopmental Alterations, Department of Speech-Language Pathology and Audiology, Bauru School of Dentistry of the University of São Paulo, Bauru, Brazil; ^2^ Association for Research in Infant and Child Development and Nelson Mandela University, Port Elizabeth, South Africa

**Keywords:** child development, child, cross-cultural comparison, neurosciences, protocols

## Abstract

**Introduction:**

Child development must be carefully evaluated, requiring assessment instruments to assess different areas of development. Griffiths Scales of Child Development 3rd Edition (Griffiths III) is used to assess different areas of development in children. This study normalized Griffiths III for the Brazilian population from 0 to 72 months.

**Methods:**

445 typically developing children from 0 to 72 months, divided into eight groups (from 0 to 6 months; 7 to 12 months; 13 to 18 months; 19 to 24 months; 25 to 36 months; 37 to 48 months; 49 to 60 months; 61 to 72 months) participated. Their tutors answered the anamnesis protocol. Denver II Developmental Screening Test and Griffiths III were applied. Statistical analysis was performed using the Mann–Whitney Test and Spearman's rank correlation coefficient. Normalization followed the criteria of the original scale.

**Results:**

There was a direct and statistically significant correlation between maternal schooling and socioeconomic status; a direct correlation in the performance between the subscales. The normalization table of Griffiths III with the developmental age of children from 0 to 72 months was elaborated through linear progression, calculated using a specific formula.

**Discussion:**

The data collected for the Brazilian population from 0 to 72 months were normalized, following the guidelines and norms of the original Griffiths III.

## 1 Introduction

Child development is complex and dynamic and influenced by numerous factors, intrinsic and extrinsic. Monitoring child development and performing early and assertive diagnosis, promoting adequate stimulation and guidance regarding the area or areas that are altered, is crucial for a good prognosis.

Focus, dedication, and continuity in studies related to normative child development are necessary. The use of assessment and diagnostic instruments, combined with the subjective clinical assessment of experienced and active professionals, enables one to carefully verify and understand the different areas of child development in a particular and individual way.

The Brazilian scenario in this regard is discouraging, considering the reduced number of standardized and normalized instruments (1–4). As an instrument for assessing and diagnosing child development, the Griffiths Scales of Child Development 3rd Edition (Griffiths III) (5) is cited, which was cross-culturally adapted to the Brazilian reality in its entirety (6).

In the international scenario, Griffiths III is widely used to monitor the typical development of children aged from 0 to 72 months, as well as to evaluate and diagnose changes in one or more areas of child development in children with suspect or diagnosed syndromes or with risk factors for changes in development (4, 7–25).

A subsequent step in the process of translation and cross-cultural adaptation of assessment instruments is the verification of psychometric properties, among which we highlight, in this study, the normalization. With normalization, it is possible to compare the performance of a child with others, considering the chronological age (26, 27). Thus, the diagnosis becomes more effective and assertive when the normalization occurs in the child's country of origin, considering the different cultures between countries.

Considering these aspects and the possibility of using Griffiths III for formal and instrumental assessment of the development of the Brazilian child population, with specific standards, this study aimed to normalize the Griffiths III for the Brazilian population aged from 0 to 72 months.

## 2 Method

The study was approved by the Committee for Ethics in Research, respecting resolution 196/96, which deals with research ethics (CAAE:84323718.3.0000.5417).

The Association for Research in Infant and Child Development (ARICD), which owns the copyright of the Griffiths Scales, authorized the study by signing a contract between the researcher, the association, and Hogrefe, the publisher of the Griffiths Scales.

### 2.1 Participants

A representative percentage of children from the Brazilian population, recruited from different cities in the State of São Paulo, participated in the study. The percentage was outlined after a statistical study considering sample calculation carried out with data from a pilot study, which had 417 participants.

The sample size calculation considered the highest standard deviation between the Griffiths III subscales (A - Foundations of Learning, B - Language and Communication, C - Eye and Hand Coordination, D – Personal, Social Emotional, E - Gross Motor) and overall performance, respecting a 95% confidence interval, using the GPower 3.1.9.2 program.

Participants were typically developing (TD) children of both sexes, aged between 0 and 72 months, representing the Brazilian population in terms of sex distribution and socioeconomic classification. These data were based on information from the Brazilian Institute of Geography and Statistics (28).

As inclusion criteria, the child needed to present typical development; be chronologically aged between 0 and 72 months; not have moderate to severe sensorineural hearing loss; not have visual impairments that would prevent the performance of the proposed procedures; show a normal result on the Denver-II Developmental Screening Test (29); and have the Free and Informed Consent Form signed by their legal guardians.

Participants were divided into eight groups to perform the sample calculation:

• Group 1 - G1: TD children aged between 0 and 6 months;
• Group 2 - G2: TD children aged between 7 and 12 months;
• Group 3 - G3: TD children aged between 13 and 18 months;
• Group 4 - G4: TD children aged between 19 and 24 months.
• Group 5 - G5: TD children aged between 25 and 36 months;
• Group 6 - G6: TD children aged between 37 and 48 months;
• Group 7 - G7: TD children aged between 49 and 60 months;
• Group 8 - G8: TD children aged between 61 and 72 months.

Considering that child development is influenced by several factors and associated with the fact that this study aims to normalize the instrument for evaluating child development, care and attention to three specific aspects, collected in the anamnesis, were necessary to contribute to the analysis of the results: sex; maternal schooling and socioeconomic status.

As for sex, the sample participants were divided into two groups: female and male.

As for the mother's level of education, an ordinal classification was performed according to the following criteria: Illiteracy (1); Incomplete Elementary School (2); Completed Elementary School (3); Incomplete High School (4); Completed High School (5); Incomplete Higher Education (6); Completed higher education (7); Postgraduate (8).

Regarding socioeconomic status, the following classification was applied, considering the number of minimum wages that the family receives monthly: Classification 1: 0–2 minimum wages; Classification 2: 2–5 minimum wages; Classification 3: above 5 minimum wages.

### 2.2 Procedures

After the signing of the Acquiescence Form by those responsible for the institutions where the data collection was carried out, contact was established with the tutors responsible for the children in the target age group of the study. The tutors who signed the Free and Informed Consent Form were invited to answer the anamnesis protocol, containing information on conditions of pregnancy, childbirth, neuropsychomotor development data, language, speech, hearing, and perception of the participant.

The Denver-II Developmental Screening Test (DDST-II) (29) was applied as an inclusion criterion in the sample, classifying the participants as typically developing children. Children who had a “Normal” result on the DDST-II were evaluated with Griffiths III (5).

The translated and adapted version of Griffiths III for the Brazilian population (6) was applied to participants aged from 0 to 72 months.

### 2.3 Data analysis

For the presentation of the data obtained during the structured interview with the tutors of the children, descriptive statistics were used with absolute and relative frequency values considering a significance level of 5% (30): Mann–Whitney Test and Spearman's rank correlation coefficient.

Normalization followed the criteria of the original Scale, through linear progression from one age group to the next (every two months), using smoothed mean and standard deviation values.

## 3 Results

Initially, 557 participants were evaluated, of which 112 presented a “Risk” result in the DDST-II, being excluded from the sample, considering the inclusion criteria. The sample consisted of 445 children, aged between 0 and 72 months, who presented a “Normal” result in the DDST-II, met the inclusion criteria, and participated in the normalization of the Griffiths III, in the Brazilian reality.

Sample calculations were performed based on the pilot study (417 participants), using the highest standard deviation value observed in group performance. The results were as follows:

G1 – 47 participants presented a higher standard deviation on subscale A with a sample calculation of 52 participants for the final sample;
G2 – 65 participants presented a higher standard deviation on subscale D with a sample calculation of 70 participants for the final sample;
G3 - 52 participants presented a higher standard deviation on subscale E with a sample calculation of 56 participants for the final sample;
G4 - 33 participants presented a higher standard deviation on subscale D with a sample calculation of 38 participants for the final sample;
G5 - 70 participants presented a higher standard deviation on subscale D with a sample calculation of 70 participants for the final sample;
G6 - 80 participants presented a higher standard deviation on subscale A with a sample calculation of 81 participants for the final sample;
G7 - 49 participants presented a higher standard deviation on subscale A with a sample calculation of 50 participants for the final sample;
G8 - 21 participants presented a higher standard deviation on subscale C with a sample calculation of 28 participants for the final sample

The sample distributed in the eight groups was analyzed in terms of sex, female and male (Table 2), and the following results were obtained: G1 - 46,2% (*n* = 24) were female and 53,8% (*n* = 28) were male; G2 – 48,6% (*n* = 34) were female and 51,4% (*n* = 36) were male; G3 - 48,2% (*n* = 27) were female and were 51,8%.(*n* = 29) male; G4 – 47,4% (*n* = 18) were female and 52,6% (*n* = 20) were male. G5 - 54,3% (*n* = 38) were female and 45,7% (*n* = 32) were male. G6 - 45,7% (*n* = 37) were female and 54,3% (*n* = 44) were male. G7 - 42% (*n* = 21) were female and 58% (*n* = 29) were male. G8 – 57,1% (*n* = 16) were female and 42,9% (*n* = 12) were male.

Table 1 presents the data on the mother's level of education, considering absolute frequency.

**Table 1:** Distribution of the sample in terms of the maternal level of education.

MLE	1	2	3	4	5	6	7	8
Group								
G1	1	4	11	27	4	5	0	0
G2	2	4	11	41	2	7	3	0
G3	4	3	13	27	5	4	0	0
G4	0	3	4	21	1	9	0	0
G5	0	0	8	9	26	4	17	6
G6	0	1	9	7	30	9	24	1
G7	0	0	7	3	17	6	13	4
G8	0	0	2	1	10	3	11	1
Total	7	15	65	136	95	47	68	12

MLE (maternal level of education); 1 (Illiteracy); 2 (Incomplete Elementary School); 3 (Completed Elementary School); 4 (Incomplete High School); 5 (Completed High School); 6 (Incomplete Higher Education); 7 (Completed Higher Education); 8 (Postgraduate); G1 (typically developing children aged between 0 and 6 months); G2 (typically developing children aged between 7 and 12 months); G3 (typically developing children aged between 13 and 18 months); G4 (typically developing children aged between 19 and 24 months); G5 (typically developing children aged between 25 and 36 months); G6 (typically developing children aged between 37 and 48 months); G7 (typically developing children aged between 49 and 60 months); G8 (typically developing children aged between 61 and 72 months).

Regarding the socioeconomic status of the sample, it was distributed considering the socioeconomic reality of the Brazilian population. The socioeconomic status was characterized according to the number of minimum wages that the family receives monthly. Below, the data are presented considering each group of the sample:

G1 - 37 participants had a family income from 0 to 2 minimum wages per month, 13 from 2 to 5, and 2 more than 5 minimum wages;
G2 - 49 participants had a family income from 0 to 2 minimum wages per month, 17 from 2 to 5, and 4 more than 5 minimum wages;
G3 - 40 participants had a family income from 0 to 2 minimum wages per month, 13 from 2 to 5, and 3 more than 5 minimum wages;
G4 - 23 participants had a family income from 0 to 2 minimum wages per month, 10 from 2 to 5, and 5 more than 5 minimum wages;
G5 - 19 participants had a family income from 0 to 2 minimum wages per month, 32 from 2 to 5, and 19 more than 5 minimum wages;
G6 - 27 participants had a family income from 0 to 2 minimum wages per month, 39 from 2 to 5, and 15 more than 5 minimum wages;
G7 - 17 participants had a family income from 0 to 2 minimum wages per month, 23 from 2 to 5, and 10 more than 5 minimum wages;
G8 - 5 participants had a family income from 0 to 2 minimum wages per month, 19 from 2 to 5, and 4 more than 5 minimum wages.

Table 2 describes the median values (50%) as well as the first quartile (25%) and third quartile (75%) of the participant's performance in the 5 subscales, after the application of the Mann–Whitney test, for comparison between sexes, considering the *p*-value statistically significant when lower than 0.05.

**Table 2:** Comparison of boys’ and girls’ performance on the 5 Griffiths III subscales.

Subscale	Sex	Median	25%	75%	*p-*value
A	M	29.5	2	61	0.796
	F	30.0	2	62	
B	M	33.0	2	62	0.773
	F	34.0	2	62	
C	M	32.5	2	67	0.727
	F	35.0	2	67	
D	M	35.0	3	64	0.733
	F	38.0	3	66	
E	M	36.0	3	63	0.974
	F	37.0	3	62	

A – Foundations of Learning Subscale; B – Language and Communication Subscale; C – Eye and Hand Coordination Subscale; D – Personal, Social Emotional Subscale; E – Gross Motor Subscale; M – male (117 participants); F – female (112 participants); 25% - first quartile; 75% - third quartile.

Table 3 describes the correlation values and the *p*-value, considered statistically significant when it was lower than 0.05, divided by group and in the total sample, for comparison between maternal education and socioeconomic status.

**Table 3:** Correlation between maternal level of education and socioeconomic status of the total sample as well as of each group individually.

Group	MLE and SS correlation	*p*-value
G1	0.452	0.001*
G2	0.363	0.002*
G3	0.477	<0.001*
G4	0.535	0.001*
G5	0.697	<0.001*
G6	0.399	<0.001*
G7	0.666	<0.001*
G8	0.489	0.008*
Total	0.559	<0.001*

MLE (maternal level of education level); SS (socioeconomic status); G1 (typically developing children aged between 0 and 6 months); G2 (typically developing children aged between 7 and 12 months); G3 (typically developing children aged between 13 and 18 months); G4 (typically developing children aged between 19 and 24 months); G5 (typically developing children aged between 25 and 36 months); G6 (typically developing children aged between 37 and 48 months); G7 (typically developing children aged between 49 and 60 months); G8 (typically developing children aged between 61 and 72 months).

* Statistically significant *p*-value, less than 0.05.

Table 4 shows the correlation between socioeconomic status and performance on the 5 subscales of Griffiths III for the eight groups. The correlation type and statistical significance were analyzed, considering a *p*-value lower than 0.05.

**Table 4:** Correlation between socioeconomic status and performance on the 5 subscales of the Griffiths III, for each group individually.

Group	Values	SS and A	SS and B	SS and C	SS and D	SS and E
G1	Correlation	−0.058	−0.060	0.042	−0.107	−0.129
	*p-*value	0.678	0.669	0.768	0.450	0.361
G2	Correlation	−0.028	−0.020	−0.069	−0.024	−0.120
	*p-*value	0.817	0.867	0.564	0.844	0.322
G3	Correlation	−0.228	−0.304	−0.264	−0.325	−0.129
	*p-*value	0.090	0.023*	0.049*	0.014*	0.343
G4	Correlation	−0.176	0.008	0.074	−0.044	0.096
	*p-*value	0.290	0.960	0.660	0.789	0.563
G5	Correlation	−0.115	−0.016	0.000	0.051	−0.021
	*p-*value	0.342	0.894	1.000	0.676	0.865
G6	Correlation	−0.009	−0.052	−0.036	−0.035	−0.175
	*p-*value	0.939	0.647	0.751	0.756	0.119
G7	Correlation	−0.093	0.028	−0.157	−0.075	−0.247
	*p-*value	0.520	0.848	0.277	0.605	0.084
G8	Correlation	0.118	0.056	0.079	0.181	0.129
	*p-*value	0.550	0.776	0.689	0.356	0.549

Caption: SS (socioeconomic status); G1 (typically developing children aged between 0 and 6 months); G2 (typically developing children aged between 7 and 12 months); G3 (typically developing children aged between 13 and 18 months); G4 (typically developing children aged between 19 and 24 months); G5 (typically developing children aged between 25 and 36 months); G6 (typically developing children aged between 37 and 48 months); G7 (typically developing children aged between 49 and 60 months); G8 (typically developing children aged between 61 and 72 months); A – Foundations of Learning Subscale; B – Language and Communication Subscale; C – Eye and Hand Coordination Subscale; D – Personal, Social Emotional Subscale; E – Gross Motor Subscale.

* Statistically significant *p*-value, less than 0.05.

Table 5 shows the correlation between the participant's performance in the 5 subscales of Griffiths III for the eight groups. The correlation type and statistical significance were analyzed, considering the *p*-value less than 0.05.

**Table 5:** Correlation between the performance in the 5 subscales of Griffiths III, of the total sample, and for each group individually.

Group	Values	A × B	A × C	A × D	A × E	B × C	B × D	B × E	C × D	C × E	D × E
G1	Correl.	0.920	0.917	0.951	0.905	0.913	0.921	0.909	0.943	0.914	0.920
	*p-*value	<0.001*	<0.001*	<0.001*	<0.001	<0.001*	<0.001*	<0.001*	<0.001*	<0.001*	<0.001*
G2	Correl.	0.866	0.871	0.839	0.749	0.876	0.867	0.784	0.852	0.747	0.841
	*p-*value	<0.001*	<0.001*	<0.001*	<0.001*	<0.001*	<0.001*	<0.001*	<0.001*	<0.001*	<0.001*
G3	Correl.	0.691	0.763	0.634	0.988	0.800	0.762	0.648	0.823	0.811	0.678
	*p-*value	<0.001*	<0.001*	<0.001*	<0.001*	<0.001*	<0.001*	<0.001*	<0.001*	<0.001*	<0.001*
G4	Correl.	0.561	0.585	0.641	0.539	0.528	0.663	0.646	0.662	0.639	0.772
	*p-*value	<0.001*	<0.001*	<0.001*	<0.001*	<0.001*	<0.001*	<0.001*	<0.001*	<0.001*	<0.001*
G5	Correl.	0.712	0.774	0.744	0.669	0.764	0.795	0.675	0.812	0.687	0.768
	*p-*value	<0.001*	<0.001*	<0.001*	<0.001*	<0.001*	<0.001*	<0.001*	<0.001*	<0.001*	<0.001*
G6	Correl.	0.881	0.836	0.739	0.807	0.808	0.774	0.832	0.718	0.795	0.791
	*p-*value	<0.001*	<0.001*	<0.001*	<0.001*	<0.001*	<0.001*	<0.001*	<0.001*	<0.001*	<0.001*
G7	Correl.	0.741	0.727	0.350	0.674	0.651	0.310	0.657	0.330	0.718	0.313
	*p-*value	<0.001*	<0.001*	<0.013*	<0.001*	<0.001*	<0.028*	<0.001*	<0.019*	<0.001*	<0.027*
G8	Correl.	0.338	0.318	0.301	0.054	0.262	0.366	0.315	0.233	0.558	0.413
	*p-*value	<0.079	<0.099	<0.120	<0.803	<0.178	<0.055	<0.134	<0.232	<0.005*	<0.045*

G1 (typically developing children aged between 25 and 36 months); G2 (typically developing children aged between 37 and 48 months); G3 (typically developing children aged between 49 and 60 months); G4 (typically developing children aged between 61 and 72 months); A – Foundations of Learning Subscale; B – Language and Communication Subscale; C – Eye and Hand Coordination Subscale; D – Personal, Social Emotional Subscale; E – Gross Motor Subscale; Correl. (correlation).

* Statistically significant *p*-value, less than 0.05.

Table 6 shows the result of the normalization of Griffiths III with the presentation of the equivalences between the Gross Results of the Subscales, the General results, and the Age of Development in the age group from 0 to 72 months.

**Table 6:** Equivalence between gross results in Griffiths III and Age of development.

Gross result	AD A	AD B	AD C	AD D	AD E
1	<15 days	<15 days	<15 days	<15 days	<15 days
2	1 m	<15 days	<15 days	<15 days	<15 days
3	2 m	1 m	1 m	1 m	1 m
4	3 m	2 m	2 m	2 m	2 m
5	4 m	3 m	3 m	3 m	3 m
6	4 ^1/3^ m	4 m	3 ^1/2^ m	4 m	3 ^1/2^ m
7	4^2/3^ m	4 ^1/2^ m	4 m	4 ^1/2^ m	4 m
8	5 m	5 m	4 ^1/2^ m	5 m	4 ^1/2^ m
9	5 ^1/3^ m	5 ^1/2^ m	5 m	5 ^1/2^ m	5 m
10	5^2/3^ m	6 m	5 ^1/2^ m	6 m	5 ^1/2^ m
11	6 m	7 m	6 m	6 ^1/2^ m	6 m
12	7 m	8 m	6 ^1/2^ m	7 m	6 ^1/2^ m
13	8 m	9 m	7 m	8 m	7 m
14	9 m	9 ^1/2^ m	8 m	8 ^1/2^ m	8 m
15	10 m	10 m	9 m	9 m	9 m
16	10 ^1/2^ m	11 m	10 m	9 ^1/2^ m	9 ^1/2^ m
17	11 m	11 ^1/3^ m	10 ^1/2^ m	10 m	10 m
18	12 m	11^2/3^ m	11 m	10 ^1/2^ m	11 m
19	13 m	12 m	11 ^1/2^ m	11 m	12 m
20	14 m	13 m	12 m	11 ^1/2^ m	12 ^1/3^ m
21	15 m	14 m	13 m	12 m	12^2/3^ m
22	16 m	14 ^1/3^ m	13 ^1/2^ m	12 ^1/2^ m	13 m
23	17 m	14^2/3^ m	14 m	13 m	14 m
24	18 m	15 m	15 m	13 ^1/2 ^m	14 ^1/4^ m
25	19 m	16 m	16 m	14 m	14^1/2^ m
26	20 m	17 m	17 m	15 m	14^3/4^ m
27	21 a 23 m	18 m	18 m	16 a 18 m	15 m
28	24 m	19 e 20 m	19 m	19 m	16 m
29	25/26 m	21 m	20 à 22 m	20 m	17 m
30	27 m	22 m	23 m	21 m	18 m
31	28 m	23 m	24 m	22 m	19 m
32	29/30 m	24 m	25 m	23m	22 m
33	31 m	25 m	25 m	24 m	21 e 22 m
34	32 m	26 m	26 m	25 m	23 m
35	33/34 m	27 m	26 m	25 m	24 m
36	35/36 m	28 m	27/28 m	26 m	25 m
37	37/38 m	29 m	29 m	27/28 m	26 m
38	39 m	30 m	30 m	29 m	27/28 m
39	40 m	31/32 m	31/32 m	30 m	29/30 m
40	41/42 m	33 m	33 m	31 m	31/32 m
41	43 m	34 m	34 m	32 m	33/34 m
42	44 m	35 m	35/36 m	33 m	35/36 m
43	45 m	36 m	37/38 m	34 m	37/38 m
44	46 m	37/38 m	39/40 m	35 m	39/40 m
45	47 m	39 m	41/42 m	36 m	41/42 m
46	48 m	40 m	43 m	37 m	43/44 m
47	49 m	41/42 m	44 m	38 m	45/46 m
48	50 m	43 m	45 m	39/40 m	47 m
49	51 m	44 m	46 m	41/42 m	48 m
50	52 m	45/46 m	47 m	43/44 m	49 m
51	53 m	47/48 m	48 m	45/46 m	50 m
52	54 m	49/50 m	49 m	47/48 m	51 m
53	55/56 m	51/52 m	50 m	49/50 m	52 m
54	57/58 m	53/54 m	51 m	51 m	53/54 m
55	59/60 m	55/56 m	52 m	52 m	55/56 m
56	61 m	57/58 m	53/54 m	53/54 m	57/58 m
57	62 m	59/60 m	55/56 m	55/56 m	59/60 m
58	63 m	61/62 m	57/58 m	57/58 m	61/62 m
59	64 m	63/64 m	59/60 m	59/60 m	63/64 m
60	65/66 m	65/66 m	61/62 m	65/66 m	65/66 m
61	67/68 m	67/68 m	63/64 m	67/68 m	67/68 m
62	69/70 m	69/70 m	65/66 m	69/70 m	69/70 m
63	71/72 m	71/72 m	67/68 m	71/72 m	71/72 m
64			69/70 m		
65			71/72 m		

AD – Age of Development; A – Foundations of Learning Subscale; B – Language and Communication Subscale; C – Eye and Hand Coordination Subscale; D – Personal, Social-Emotional Subscale; E – Gross Motor Subscale.

## 4 Discussion

Studies aiming to normalize an assessment instrument require a robust methodology that accounts for variables potentially affecting the instrument's application results. Thus, before the application, the instrument is adapted or prepared in a manner consistent with the culture of the population to which it will be applied (2, 31, 32).

Griffiths III was translated and cross-culturally adapted into the Brazilian Portuguese language (6), following a rigorous and careful process, based on the methodology proposed by Beaton (31).

Many countries use the Griffiths scales, such as Germany, South Africa, East Africa, Australia, Belgium, Cambodia, China, Philippines, France, Spain, Greece, Netherlands, Italy, Macedonia, Malaysia, Portugal, Sweden, Switzerland, Russia, and Thailand.

The establishment of the number of participants in the sample respected the sample calculation based on the highest standard deviation verified, based on the performance of the participants in each subscale, and also on the general performance. This calculation was performed for each of the eight groups into which the sample was divided, considering the age groups. The confidence interval respected in the sample calculation was 95%.

Sampling is a highly developed and sophisticated field of statistics that studies the research planning technique. It enables inferences about a universe from the study of part of its components. As sampling seeks the inference from a strictly statistical point of view, the procedure will only be valid if the sample is representative of the population. The sample calculation aims to reduce the costs and time of the estimation process, maintaining a safe minimum precision (33).

Regarding sex, maternal level of education (Table 1), and socioeconomic status, the distributions of the participants were presented. These factors were selected and included in the data analysis, considering the analyses performed in the Griffiths III Manual (5). Studies with GDMS also considered these variables (4, 9, 34–37).

DDST-II is a screening test widely used to screen children aged from 0 to 72 months, with “Normal” or “Risk” results. Due to its wide application and use by our research group, and also for its recognition in the scientific, clinical, and research community, this was the instrument chosen to select the sample (38–40).

Two hundred and fifteen girls and two hundred and thirty boys were evaluated. There was no statistically significant difference in performance between girls and boys (Table 2), analyzing the performance in each Griffiths III subscale individually. There are studies in the literature that corroborate this finding (5, 9, 34).

When analyzing the correlation between the maternal level of education and socioeconomic status (Table 3), there was a direct and statistically significant correlation in the eight individual groups and, therefore, in the total sample. According to the IBGE's Synthesis of Social Indicators (2010), the two correlated variables are directly proportional, which shows the possibility of considering only one of them in further analyses. Thus, socioeconomic status was chosen to analyze its correlation with participants' performance in the Griffiths III subscales.

Table 4 shows the correlation between SS and the performance of the participants in the five subscales of Griffiths III (A, B, C, D, and E), for each group individually (G1, G2, G3, G4, G5, G6, G7 and G8). A statistically significant correlation was observed between SS and the performance in the subscales B, C, and D, only for G3 (from 13 to 18 months). The correlations were weak, some being direct and others inverse. It is noteworthy that only in group 5 (from 25 to 36 months), for SS and Eye and Hand Coordination, there was no correlation. Group 8 (from 61 to 72 months) was the only group that showed a direct correlation between SS and all subscales (A, B, C, D, and E). Groups 2, 3, and 6 (from 7 to 12; 13 to 24; and 37 to 48 months) showed an indirect correlation between SS and all subscales. There was heterogeneity regarding the correlation of SS and performance on the subscales, according to the age group of the participants.

The result of the statistical analysis regarding the correlation between SS and the performance of the participants is different when compared to some studies in the literature (7, 41).

Considering the aim of the study of normalizing an assessment instrument, the sample was not distributed homogeneously in the three levels of socioeconomic classification analyzed (1, 2, and 3), which differs from studies found in the literature that distributed the sample evenly among socioeconomic levels, enabling the analysis of the interference of this factor in the participants' performance.

The correlation between the performance of the participants in the five subscales, among themselves, for the eight age groups, was analyzed (Table 5). A direct correlation was found between all subscales, in the eight groups, as well as in the total sample. In groups 1; 2; 3; 5 and 6 (from 0 to 6; 7 to 12; 13 to 18; 25 to 36; and 37 to 48 months) individually, all correlations were strong, direct, and statistically significant.

There was a direct and statistically significant correlation between all subscales of group 4 (from 19 to 24 months), although this correlation varies between 0.528 and 0.663, in performance comparisons between subscales, not indicating as strong a correlation as in the other analyses.

In group 7 (from 49 to 60 months), strong correlations were observed between subscales A (Foundations of Learning), B (Language and Communication), C (Eye and Hand Coordination), and E (Gross Motor). When subscale D (Personal, Social Emotional) was analyzed with the other four subscales, the correlations were weak, despite being direct and statistically significant.

Correlations in group 8 (from 61 to 72 months) were weak and significant only for subscale C (Eye and Hand Coordination) and (Gross Motor) and for subscale D (Personal, Social-Emotional) and E (Gross Motor). Group 8 showed the lowest standard deviation in the statistical calculation. Thus, it was the one with the lowest number of participants in the sample. This fact may have influenced the result of the correlation, in addition to emphasizing the need for a thorough analysis of which activities/skills were expected in this age group, since it is configured in the last age group of Griffiths III.

The literature describes the influence of the performance of a development area on other areas (34, 42–44). The importance of early diagnosis to assess alteration in one or more areas of child development so that it is possible to provide essential stimulation to the child, preventing negative interference in other areas that are not altered, is unanimous (3, 20, 22, 37, 45–47).

Statistical analysis for data normalization (Table 6) was applied, in which the Child's Age of Development was provided as a result, through the linear progression of an age group for the next (every two months), using smoothed mean and standard deviation values, calculated from a specific formula used in the original Scale (3). The raw data from the normalization process, as well as the child's developmental age (Table 6) will be made available to the statistician responsible for the statistical analysis of the original scale, who will process the data to prepare the other results for the development of the scale in the Brazilian Portuguese version.

In the final normalization of the Griffiths III reference tables, reference values of the Scaled Index, Development Quotient with 95% Confidence Interval, Stanine, and Percentile will be generated, all associated and interconnected.

This process will occur concurrently with the period of translation of the Griffiths III Manuals into Brazilian Portuguese and other bureaucratic procedures so that in a not-distant future the instrument for the application of the Griffiths III can be made available for use in Brazil.

## 5 Conclusion

The normalization of the data collected for the Brazilian population aged from 0 to 72 months was performed, following the guidelines and norms of the Griffiths III. Soon, Brazilian health professionals who work with children will have the opportunity to assess child development using this scale, diagnosing global changes in child development or specific areas. The application of the scale and the analysis of the results, appropriately, combined with clinical evaluation by an experienced professional, will provide assertive early diagnoses and essential stimulation, prioritizing the period of greatest brain plasticity in the child.

## Data Availability

The raw data supporting the conclusions of this article will be made available by the authors, without undue reservation.
